# Activation of AMPK inhibits TGF-β1-induced airway smooth muscle cells proliferation and its potential mechanisms

**DOI:** 10.1038/s41598-018-21812-0

**Published:** 2018-02-26

**Authors:** Yilin Pan, Lu Liu, Shaojun Li, Ke Wang, Rui Ke, Wenhua Shi, Jian Wang, Xin Yan, Qianqian Zhang, Qingting Wang, Limin Chai, Xinming Xie, Manxiang Li

**Affiliations:** 1 Department of Respiratory and Critical Care Medicine, the First Affiliated Hospital of Xi’an Jiaotong University, Xi’an, 710061 P.R. China; 2 School of Pharmacy, Xi’an Jiaotong University, Xi’an, 710061 P.R. China

## Abstract

The aims of the present study were to examine signaling mechanisms underlying transforming growth factor β1 (TGF-β1)-induced airway smooth muscle cells (ASMCs) proliferation and to determine the effect of adenosine monophosphate-activated protein kinase (AMPK) activation on TGF-β1-induced ASMCs proliferation and its potential mechanisms. TGF-β1 reduced microRNA-206 (miR-206) level by activating Smad2/3, and this in turn up-regulated histone deacetylase 4 (HDAC4) and consequently increased cyclin D1 protein leading to ASMCs proliferation. Prior incubation of ASMCs with metformin induced AMPK activation and blocked TGF-β1-induced cell proliferation. Activation of AMPK slightly attenuated TGF-β1-induced miR-206 suppression, but dramatically suppressed TGF-β1-caused HDAC4 up-expression and significantly increased HDAC4 phosphorylation finally leading to reduction of up-regulated cyclin D1 protein expression. Our study suggests that activation of AMPK modulates miR-206/HDAC4/cyclin D1 signaling pathway, particularly targeting on HDAC4, to suppress ASMCs proliferation and therefore has a potential value in the prevention and treatment of asthma by alleviating airway remodeling.

## Introduction

Asthma is a chronic inflammatory airway disease characterized by airway hyperresponsiveness and airway remodeling^1^. Progressive airway remodeling finally leads to irreversible airflow obstruction which is a significant feature of severe asthma^2^. Pathologic changes of airway remodeling include airway smooth muscle cells (ASMCs) hypertrophy/proliferation/migration, subepithelial fibrosis and epithelial alterations^3^. Among these, ASMCs proliferation is believed to play a critical role in the development of airway remodeling. Thus, exploring the mechanisms underlying ASMCs proliferation and investigating appropriate targets are meaningful for the prevention and treatment of airway remodeling and management of asthma.

Transforming growth factor β1 (TGF-β1) has been shown to be elevated in airway, peripheral blood and bronchoalveolar lavage fluid (BALF) in asthmatic patients^4–6^, which stimulates ASMCs proliferation^7^. However, the mechanisms underlying TGF-β1-induced ASMCs proliferation are still largely unclear. MicroRNAs (miRNAs) are single-stranded 21-22-nucleotide noncoding RNAs which are able to regulate gene expression at a post-transcriptional level by blocking the translation or promoting the degradation of target gene mRNAs^8^. It has been reported that miRNAs play important roles in various physiological and pathological processes, including cell metabolism, differentiation, apoptosis and proliferation^9^. MiR-206 has been shown to be related to cell proliferation, which is down-regulated in various types of cancer cells. Overexpression of miR-206 inhibits proliferation of cancer cells and pulmonary artery smooth muscle cells^10–12^, and reduction of miR-206 has been further indicated in the lung and blood of patients with asthma^13^. In addition, TGF-β1 has been shown to inhibit myogenic differentiation through down-regulation of miR-206 in myoblasts^14^. Therefore, it is interesting to know whether miR-206 mediates TGF-β1-induced ASMCs proliferation.

Adenosine monophosphate-activated protein kinase (AMPK) is a metabolic sensor, which is activated by the increase of AMP/ATP ratio caused by hypoxia, ischemia and heat shock, or other stimuli independent of energy crisis such as chemical compounds^15,16^. Activation of AMPK also regulates various cellular processes including cell proliferation, apoptosis and migration^17^. Recent studies have shown that activation of AMPK reduces TGF-β1-induced cell proliferation, differentiation, migration and epithelial-to-mesenchymal transition in different types of cells, such as myofibroblasts, mesothelial cells and cancer cells^18–20^. However, it is still unknown whether activation of AMPK suppresses TGF-β1-induced ASMCs proliferation and its potential mechanisms. To address these issues, miR-206 expression and its upstream regulator and downstream targets were examined in primary cultured ASMCs stimulated with TGF-β1. The effect of AMPK activation on TGF-β1-induced ASMCs proliferation and its mechanisms were also explored.

## Results

### TGF-β1 stimulates ASMCs proliferation via activation of Smad2/3

**Figure 1:**
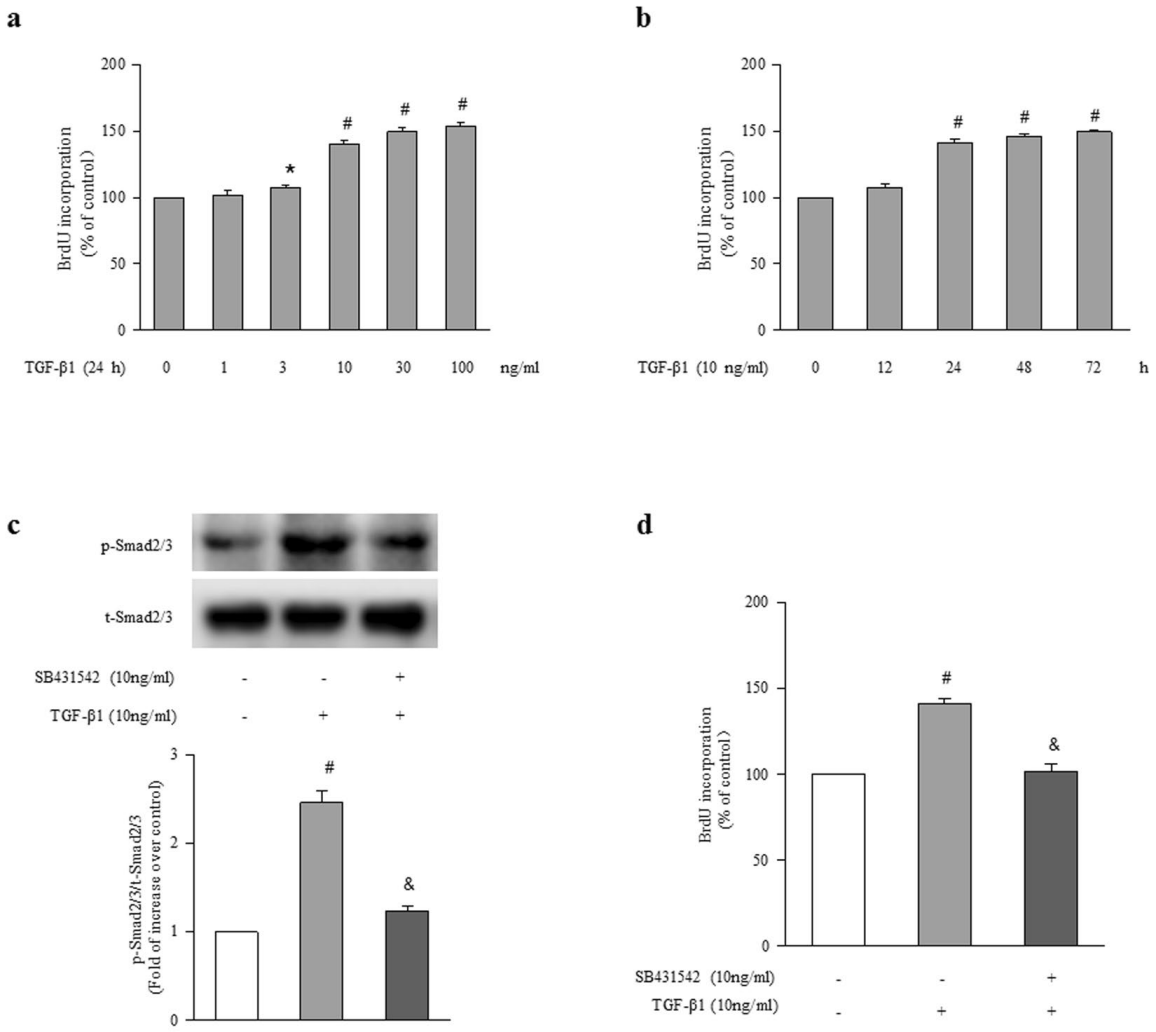
TGF-β1 stimulates ASMCs proliferation via activation of Smad2/3. (**a**) ASMCs were stimulated with different concentrations of TGF-β1 ranging from 0 to 100 ng/ml for 24 h, the rate of BrdU incorporation in cells was determined by BrdU ELISA assay Kit (n = 4 per group). (**b**) Cells were exposed to 10 ng/ml TGF-β1 for the indicated times, BrdU incorporation in cells was measured (n = 4 per group). (**c**) ASMCs were treated with SB431542 (10 μM) for 1 h before stimulation with TGF-β1 (10 ng/ml) for 1 h, the phosphorylation of Smad2/3 was determined by immunoblotting (n = 4 per group). The full-length blots of Fig. 1c are presented in Supplementary Fig. S1. (**d**) ASMCs were treated with SB431542 (10 μM) for 1 h and then stimulated with TGF-β1 (10 ng/ml) for 24 h, BrdU incorporation in cells was measured (n = 4 per group). *P < 0.05 versus control. ^#^P < 0.01 versus control. ^&^P < 0.01 versus TGF-β1-treated cells.

To examine the effect of TGF-β1 on ASMCs proliferation, cells were treated with different concentrations of TGF-β1 (0, 1, 3, 10, 30, 100 ng/ml) for different times (0, 12, 24, 48, 72 h), and cell proliferation was determined using BrdU incorporation assay. Figure 1a shows that TGF-β1 dose-dependently stimulated ASMCs proliferation, and 10 ng/ml TGF-β1 triggered a 1.40-fold increase in BrdU incorporation in 24 h compared with control (P < 0.01). Figure 1b demonstrates that TGF-β1 stimulated ASMCs proliferation in a time-dependently manner, and 10 ng/ml TGF-β1 caused a 1.50-fold increase in BrdU incorporation over control at the time of 72 h (P < 0.01). These results indicate that TGF-β1 effectively stimulates ASMCs proliferation.

To explore the signaling mechanisms underlying TGF-β1-induced ASMCs proliferation, the phosphorylation of Smad2/3 was determined using immunoblotting. Figure 1c shows that treatment of cells with TGF-β1(10 ng/ml) for 1 h increased Smad2/3 phosphorylation to 2.46-fold compared to control (P < 0.01), while prior treatment of cells with SB431542 (10 μM), an ALK5/Smad2/3 inhibitor, for 1 h suppressed TGF-β1-stimulated Smad2/3 phosphorylation, which declined to 1.23-fold over control (P < 0.01 versus TGF-β1-treated cells). Figure 1d indicates that the presence of SB431542 dramatically suppressed TGF-β1 (10 ng/ml, 24 h)-induced ASMCs proliferation, the rate of BrdU incorporation declined from 1.41-fold over control to 1.02-fold over control (P < 0.01). These results suggest that activation of Smad2/3 specifically medicates TGF-β1-induced ASMCs proliferation.

### Smad2/3 mediates TGF-β1-induced miR-206 down-regulation, HDAC4 and cyclin D1 up-regulation in ASMCs

**Figure 2:**
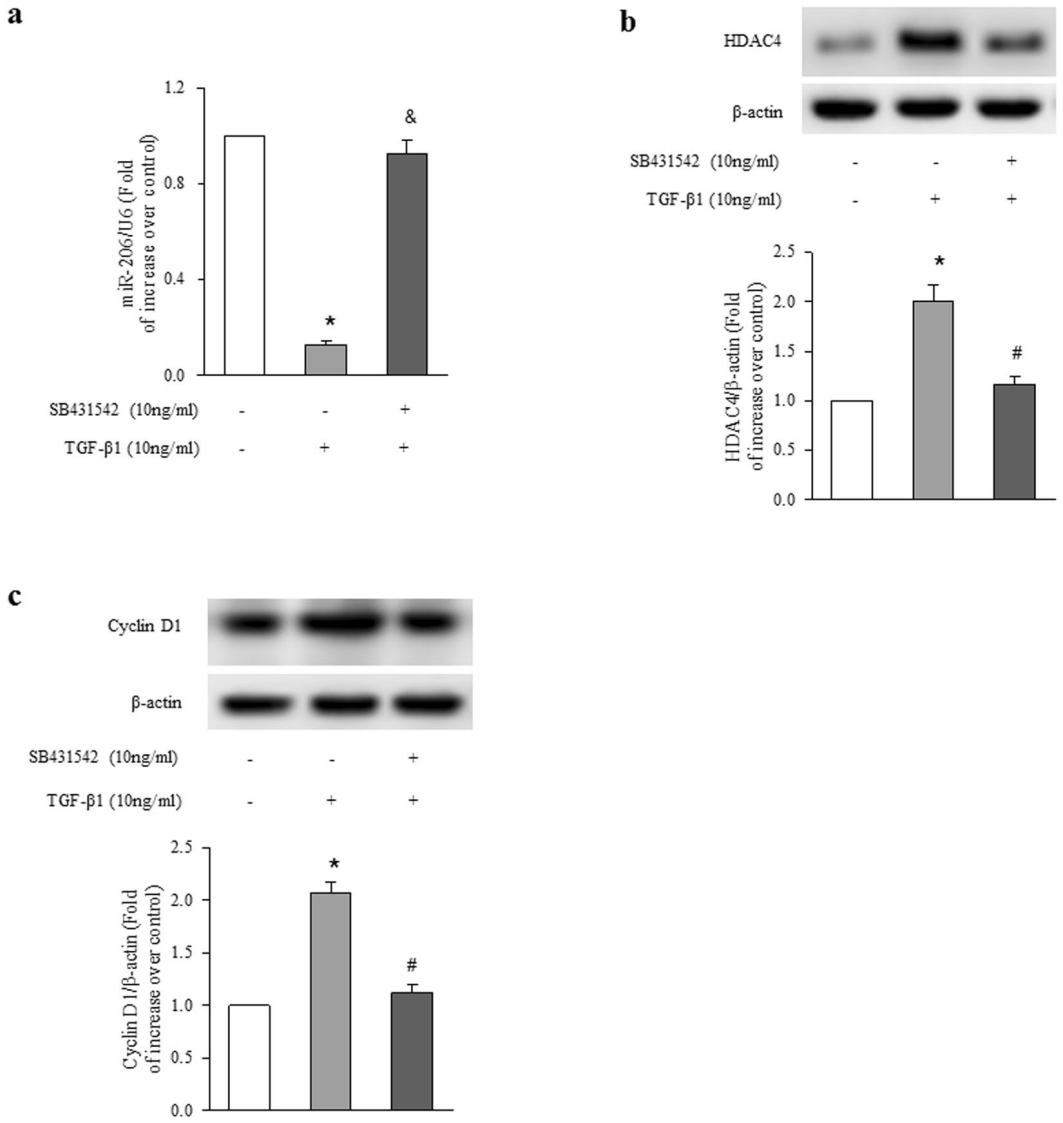
Smad2/3 mediates TGF-β1-induced alterations of miR-206, HDAC4 and cyclin D1. ASMCs were treated with SB431542 (10 μM) for 1 h before stimulation with TGF-β1 (10 ng/ml) for 24 h. (**a**) The level of miR-206 was measured using qRT-PCR. U6 small nuclear RNA served as a loading control (n = 4 per group). (**b**) HDAC4 protein level was analyzed using immunoblotting (n = 4 per group). (**c**) Cyclin D1 protein level was determined using immunoblotting (n = 4 per group). The full-length blots of Fig. 2b and 2c are presented in Supplementary Fig. S1. *P < 0.01 versus control. ^#^P < 0.01 versus TGF-β1-treated cells.

To investigate whether TGF-β1 down-regulates miR-206 expression and its mechanisms in ASMCs, cells were stimulated with 10 ng/ml TGF-β1 for 24 h with or without prior treatment with SB431542 (10 μM) for 1 h, the level of miR-206 was determined using qRT-PCR. As shown in Fig. 2a, TGF-β1 reduced mature miR-206 level to 0.13-fold over control (P < 0.01 versus control), while the presence of SB431542 dramatically blocked TGF-β1-induced miR-206 reduction, which raised to 0.92-fold over control (P < 0.01 versus TGF-β1 treated cells). These results suggest that activation of Smad2/3 particularly mediates TGF-β1 down-regulation of miR-206 in ASMCs.

We next examined whether TGF-β1 signaling regulates protein expression of HDAC4 and cyclin D1 contributing to ASMCs proliferation. Cells were treated with 10 μM SB431542 for 1 h and then stimulated with TGF-β1 (10 ng/ml) for 24 h. Figure 2b indicates that treatment of ASMCs with TGF-β1 increased HDAC4 protein level to 2.01-fold over control (P < 0.01), while pretreatment of cells with SB431542 blocked TGF-β1-induced HDAC4 protein expression, which declined to 1.16-fold over control (P < 0.01 versus TGF-β1-treated cells). Figure 2c shows that TGF-β1 stimulation resulted in a 2.06-fold increase in cyclin D1 protein level compared to control (P < 0.01), while the presence of SB431542 reduced TGF-β1-induced cyclin D1 protein level to 1.12-fold over control (P < 0.01 versus TGF-β1-treated cells). These results suggest that TGF-β1 up-regulates HDAC4 and cyclin D1 protein expression in ASMCs by activating Smad2/3 cascade.

### miR-206 regulates HDAC4/cyclin D1 expression in ASMCs

**Figure 3:**
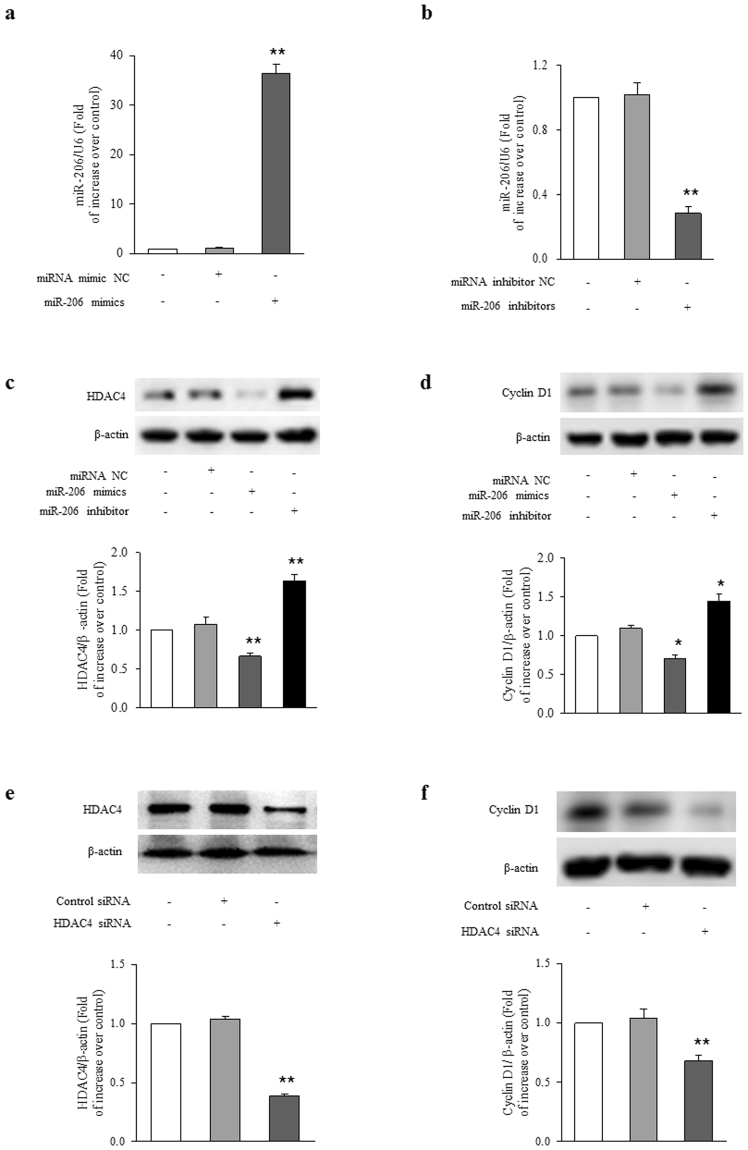
miR-206 regulates HDAC4/cyclin D1 expression in ASMCs. (**a**) ASMCs were transfected with miR-206 mimics or miR-NC for 48 h, the expression of miR-206 was examined by qRT-PCR. U6 small nuclear RNA served as a loading control (n = 4 per group). (**b**) ASMCs were transfected with miR-206 inhibitors or miR-NC for 48 h, the level of miR-206 was examined by qRT-PCR. U6 small nuclear RNA served as a loading control (n = 4 per group). ASMCs were transfected with miR-206 mimics, miR-206 inhibitors or miR-NC for 48 h. (**c**) HDAC4 protein level were analyzed using immunoblotting (n = 4 per group). (**d**) Cyclin D1 protein level was determined using immunoblotting (n = 4 per group). (**e**) ASMCs were transfected with sequence-specific HDAC4 siRNA or non-targeting siRNA for 48 h, HDAC4 protein level was examined using immunoblotting (n = 4 per group). (**f**) ASMCs were transfected with sequence-specific HDAC4 siRNA or non-targeting siRNA for 48 h, cyclin D1 protein level was determined by immunoblotting (n = 4 per group). The full-length blots of Fig. 3c–f are presented in Supplementary Fig. S2. *P < 0.05 versus control. **P < 0.01 versus control.

To investigate whether miR-206 specifically regulates HDAC4 and cyclin D1 expression in ASMCs, transfection of miRNA negative control (miRNA NC), miR-206 mimics, or miR-206 inhibitor were applied in the study. Figure 3a shows that transfection of ASMCs with miR-206 mimics for 48 h resulted in a 36-fold increase in miR-206 level compared to control (P < 0.01), and transfection of cells with miR-206 inhibitor reduced the miR-206 level to 28% of control (Fig. 3b, P < 0.01), whereas miRNA NC transfection did not change miR-206 level. As shown in Fig. 3c, transfection of ASMCs with miR-206 mimics for 48 h reduced HDAC4 protein level to 0.67-fold over control (P < 0.01), while transfection of ASMCs with miR-206 inhibitors caused a 1.64-fold increase in HDAC4 protein level compared to control (P < 0.01). Figure 3d shows that transfection of cells with miR-206 mimics reduced cyclin D1 protein level to 0.71-fold over control (P < 0.05), while transfection of ASMCs with miR-206 inhibitors induced a 1.45-fold increase in cyclin D1 protein level compared to control (P < 0.05). These results indicate that miR-206 regulates HDAC4 and cyclin D1 protein expression in ASMCs.

**Figure 4:**
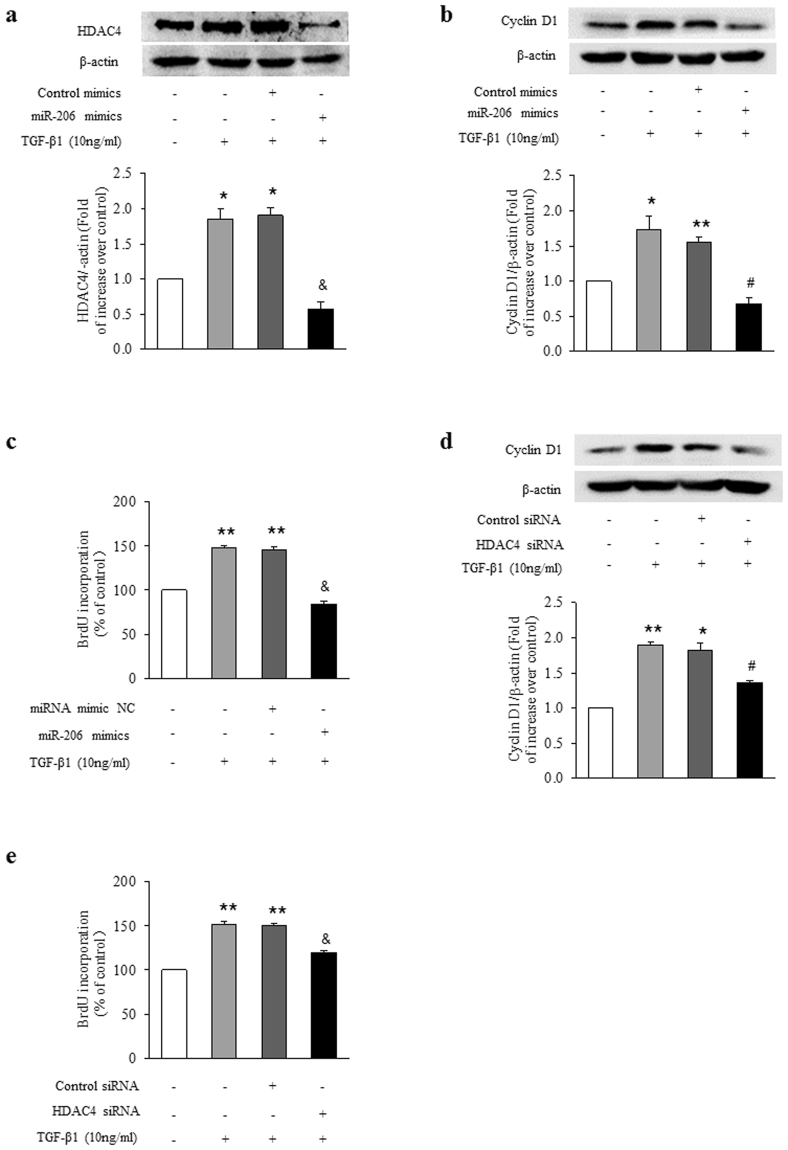
Up-regulation of HDAC4 and cyclin D1 by miR-206 reduction mediates TGF-β1-induced ASMCs proliferation. ASMCs were transfected with miR-206 mimics or miR-NC for 24 h and then stimulated with 10 ng/ml TGF-β1 for 24 h. (**a**) HDAC4 protein level was analyzed using immunoblotting (n = 4 per group). (**b**) Cyclin D1 protein level was determined by immunoblotting (n = 4 per group). (**c**) BrdU incorporation rate was measured (n = 4 per group). ASMCs were transfected with sequence-specific HDAC4 siRNA or non-targeting siRNA for 24 h and then stimulated with 10 ng/ml TGF-β1 for 24 h. (**d**) Cyclin D1 protein level was determined by immunoblotting (n = 4 per group). The full-length blots of Fig. 4a, 4b and 4d are presented in Supplementary Fig. S3. (**e**) BrdU incorporation rate was measured (n = 4 per group). *P < 0.05 versus control. **P < 0.01 versus control. ^#^P < 0.05 versus TGF-β1-treated cells. ^&^P < 0.01 versus TGF-β1-treated cells.

To further explore whether loss of HDAC4 specifically mediates cyclin D1 reduction, HDAC4 was first silenced with sequence specific siRNA and then cyclin D1 protein level was measured. As shown in Fig. 3e, transfection of ASMCs with sequence specific siRNA-HDAC4 for 48 h significantly reduced HDAC4 protein level to 38% of control (P < 0.01), whereas non-targeting siRNA did not change the HDAC4 protein level. Loss of HDAC4 by siRNA silencing reduced cyclin D1 protein level to 0.68-fold over control (Fig. 4f; P < 0.01). These results suggest that HDAC4 positively regulates cyclin D1 protein level in ASMCs.

### Up-regulation of HDAC4 and cyclin D1 by miR-206 reduction mediates TGF-β1-induced ASMCs proliferation

To determine whether reduction of miR-206 specifically mediates TGF-β1-induced up-regulation of HDAC4 and cyclin D1 and therefore ASMCs proliferation, cells were treated with 10 ng/ml TGF-β1 for 24 h with or without prior overexpression of miR-206 for 24 h. Figure 4a indicates that prior overexpression of miR-206 reversed TGF-β1-induced HDAC4 protein elevation, which decreased from 1.85-fold over control to 0.57-fold over control (P < 0.01). Figure 4b shows that TGF-β1 stimulation caused a 1.73-fold increase in cyclin D1 protein level compared to control (P < 0.05), while overexpression of miR-206 reduced TGF-β1-induced cyclin D1 protein level to 0.68-fold over control (P < 0.05 versus TGF-β1-stimulated cells). As shown in Fig. 4c, overexpression of miR-206 significantly reversed TGF-β1-triggered ASMCs proliferation, the BrdU incorporation rate reduced from 1.48-fold over control to 0.84-fold over control (P < 0.01). These results suggest that loss of miR-206 mediated TGF-β1-induced HDAC4 and cyclin D1 up-regulation and ASMCs proliferation.

To determine whether elevation of HDAC4 caused by miR-206 down-regulation mediates TGF-β1 induced cyclin D1 expression and ASMCs proliferation, cells were treated with 10 ng/ml TGF-β1 for 24 h with or without prior silencing of HDAC4 for 24 h and then cyclin D1 protein level and cell proliferation were measured. Transfection efficiency of siRNA-HDAC4 was shown in Fig. 3e. As shown in Fig. 4d, knock down of HDAC4 reversed TGF-β1-induced cyclin D1 induction, which decreased from 1.89-fold over control to 1.36-fold over control (P < 0.05). Figure 4e shows that loss of HDAC4 suppressed TGF-β1-stimulated ASMCs proliferation, the BrdU incorporation rate reduced from 1.52-fold over control to 1.19-fold over control (P < 0.01). Non-targeting siRNA did not affect cyclin D1 protein level and cell proliferation. These results suggest that miR-206/HDAC4/cyclin D1 pathway specifically mediates TGF-β1 stimulation of ASMC proliferation.

### Activation of AMPK by metformin inhibits TGF-β1-stimulated ASMCs proliferation

**Figure 5:**
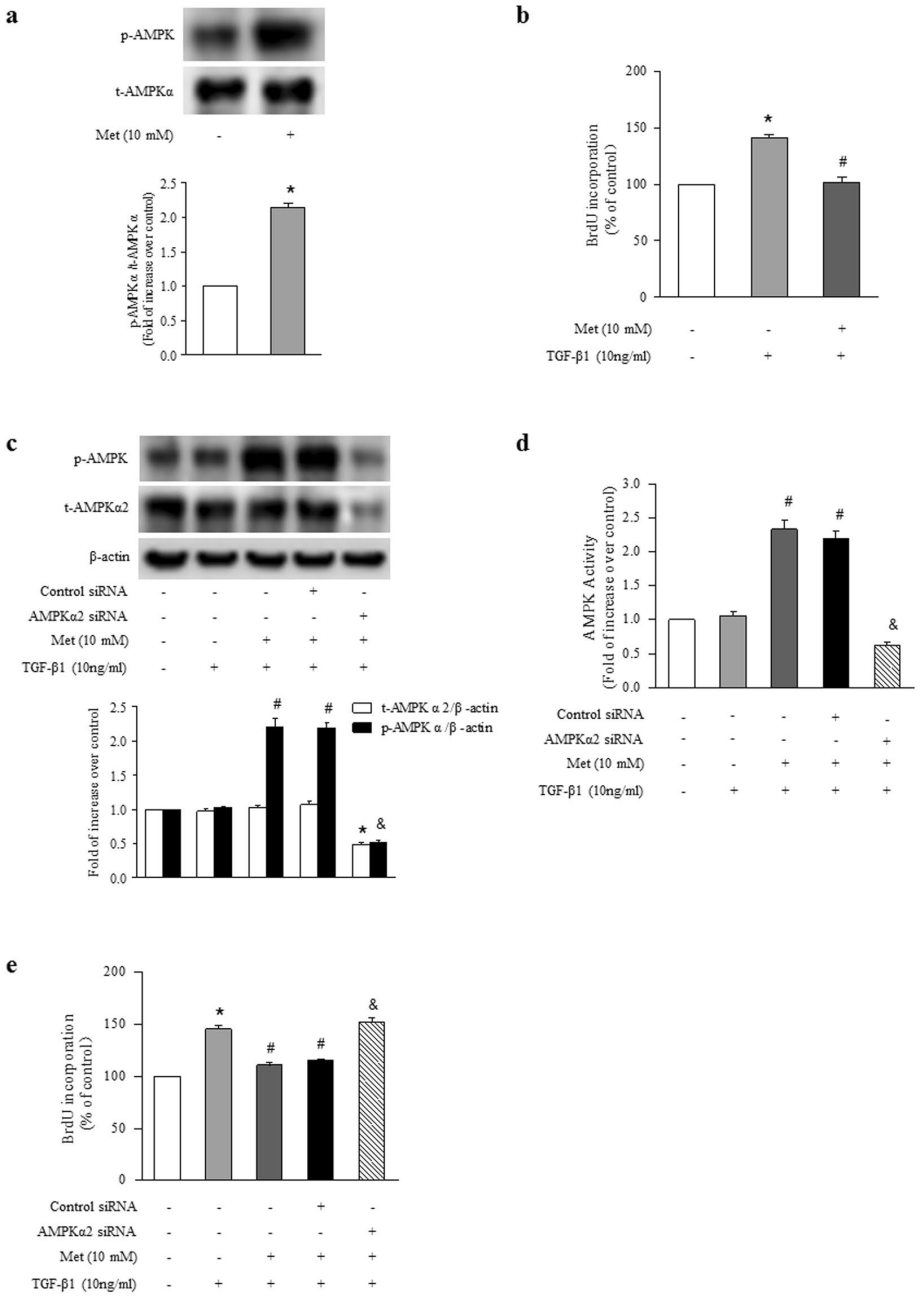
Activation of AMPK by metformin inhibits TGF-β1-induced ASMCs proliferation. (**a**) ASMCs were treated with metformin (10 mM) for 6 hours, the phosphorylation of AMPK was measured by immunoblotting (n = 4 per group). (**b**) ASMCs were treated with metformin (10 mM) for 6 h before stimulation with TGF-β1 (10 ng/ml) for 24 h, BrdU incorporation was measured (n = 4 per group). ASMCs were transfected with AMPK α2-specific or non-targeting siRNA for 48 h, and then treated with metformin (10 mM) for 6 h before stimulation with TGF-β1 (10 ng/ml) for 1 h. (**c**) Phosphorylation of AMPK α was determined by immunoblotting (n = 4 per group). The full-length blots of Fig. 5a and 5c are presented in Supplementary Fig. S4. (**d**) AMPK activity was assessed using AMPK activity kit (n = 4 per group). (**e**) ASMCs were transfected with AMPK α2-specific or non-targeting siRNA for 24 h, and then treated with metformin (10 mM) for 6 h before stimulation with TGF-β1 (10 ng/ml) for 24 h. The rate of BrdU incorporation was detected (n = 4 per group). *P < 0.01 versus control. ^#^P < 0.01 versus TGF-β1-treated cells. ^&^P < 0.01 versus metformin and TGF-β1-stimulated cells.

To examine the effect of activation of AMPK on TGF-β1 induced ASMCs proliferation, metformin was used to activate AMPK. Figure 5a shows that metformin (10 mM, 6 h) increased the phosphorylation of AMPK α to 2.14-fold compared with control cells (P < 0.01). Pretreatment of cells with metformin (10 mM) for 6 h suppressed TGF-β1 (10 ng/ml, 24 h)-induced ASMCs proliferation, BrdU incorporation rate dropped from a 1.43-fold increase over control to a 1.08-fold increase over control (Fig. 5b, P < 0.01).

To verify AMPK activation mediates metformin inhibition of TGF-β1 induced ASMCs proliferation, AMPK α2 was silenced in the study. Figure 5c indicates that knockdown of AMPK α2 reduced metformin (10 mM, 6 h)-induced AMPK α phosphorylation in the presence of TGF-β1, which declined from a 2.21-fold increase over control to a 0.52-fold increase over control (P < 0.01). Similarly, metformin-induced AMPK activity was also decreased from a 2.33-fold increase over control in metformin and TGF-β1 co-treated cells to a 0.63-fold increase over control in cells lacking AMPK α2 in the presence of metformin and TGF-β1 (Fig. 5d, P < 0.01). Figure 5e shows that loss of AMPK abolished metformin on TGF-β1-stimulated cell proliferation, BrdU incorporation rate was raised from 1.11-fold over control in metformin and TGF-β1-treated cells to 1.52-fold over control in cells lacking AMPK α2 in the presence of metformin and TGF-β1 (P < 0.01). These results suggest that the activation of AMPK mediates metformin inhibition of TGF-β1-induced ASMCs proliferation.

### Molecular mechanisms underlying AMPK inhibition of TGF-β1 induced ASMCs proliferation

**Figure 6:**
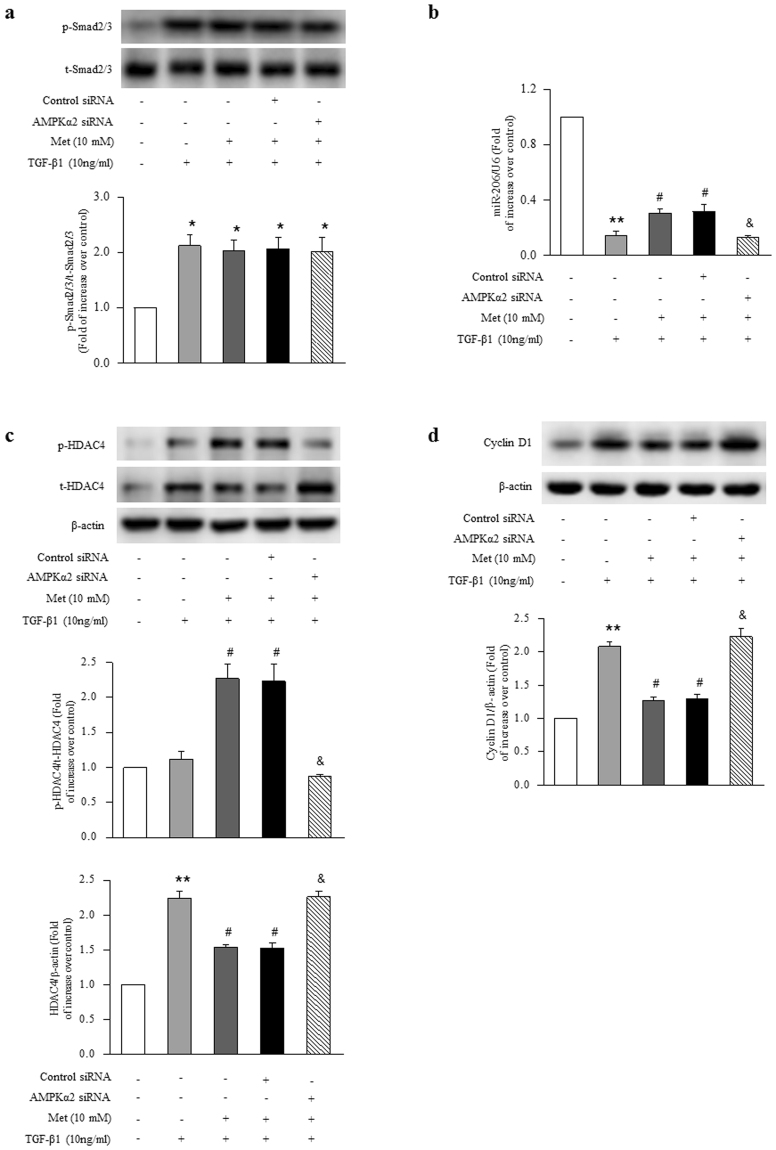
The mechanisms underlying activation of AMPK inhibition of TGF-β1-induced ASMCs proliferation. (**a**) ASMCs were transfected with AMPK α2-specific or non-targeting siRNA for 48 h, and then treated with metformin (10 mM) for 6 h before stimulation with TGF-β1 (10 ng/ml) for 1 h. The phosphorylation of Smad2/3 was determined by immunoblotting (n = 4 per group). ASMCs were transfected with AMPK α2-specific or non-targeting siRNA for 24 h, and then treated with metformin (10 mM) for 6 h before stimulation with TGF-β1 (10 ng/ml) for 24 h. (**b**) The expression of miR-206 was examined by qRT-PCR (n = 4 per group). (**c**) The expression and phosphorylation of HDAC4 were analyzed using immunoblotting (n = 4 per group). (**d**) Cyclin D1 protein level was determined using immunoblotting (n = 4 per group). The full-length blots of Fig. 6a, 6c and 6d are presented in Supplementary Fig. S4. *P < 0.05 versus control. **P < 0.01 versus control. ^#^P < 0.01 versus TGF-β1-treated cells. ^&^P < 0.01 versus metformin and TGF-β1-stimulated cells.

To determine the major target of AMPK regulation of TGF-β1-induced ASMCs proliferation, we first examined whether AMPK affects the phosphorylation of Smad2/3. Figure 6a demonstrates that pretreatment of cells with metformin did not affect TGF-β1-induced the phosphorylation of Smad2/3, suggesting that AMPK did not target on Smad2/3. Next, we investigated whether AMPK works through miR-206. As shown in Fig. 6b, prior treatment of cells with metformin (10 mM) slightly increased TGF-β1-induced (10 ng/ml) miR-206 reduction, which raised from 0.14-fold over control to 0.31-fold over control (P < 0.01), while loss of AMPK α2 abolished the effect of metformin on miR-206, which declined to 0.13-fold over control again (P < 0.01 versus metformin and TGF-β1-treated cells), suggesting that miR-206 might not be the major target of AMPK.

We further examined whether AMPK functions via HDAC4. Due to the previous findings that phosphorylation of HDAC4 leads to HDAC4 inactivation^21–23^, we determined HDAC4 expression and phosphorylation in the present study. Figure 6c shows that activation of AMPK by metformin inhibited TGF-β1-induced HDAC4 protein expression, which declined from a 2.25-fold increase over control to a 1.54-fold increase over control (P < 0.01). Activation of AMPK by metformin also increased the phosphorylation of HDAC4, which raised from a 1.12-fold increase over control in cells treated with TGF-β1 for 24 h to a 2.27-fold increase over control in cells pre-treated with metformin for 6 h and then stimulated with TGF-β1 for 24 h (P < 0.01). Deletion of AMPK α2 reversed the inhibitory effect of metformin on HDAC4 protein expression, which increased to 2.26-fold over control again (P < 0.01 versus metformin and TGF-β1-treated cells), and blocked metformin-induced HDAC4 phosphorylation, which declined to 0.88-fold over control (P < 0.01 versus metformin and TGF-β1-treated cells). These results suggest that activation of AMPK might simultaneously regulate HDAC4 expression and phosphorylation to inhibit ASMCs proliferation. Accompanied with the changes of HDAC4 expression and phosphorylation by metformin, TGF-β1-induced cyclin D1 protein up-expression was also blocked by metformin, which declined from 2.08-fold over control to 1.27-fold over control (P < 0.01), whereas deletion of AMPK α2 reversed the inhibitory effect of metformin on cyclin D1 protein level, which increased to 2.23-fold again compared with control (P < 0.01 versus metformin and TGF-β1-treated cells). These results suggest that activation of AMPK by metformin inhibits TGF-β1-induced ASMCs proliferation by suppressing miR-206/HDAC4/cyclin D1 pathway, especially targeting on HDAC4.

## Discussion

In the present study, we have shown that TGF-β1 stimulates ASMCs proliferation by Smad2/3 mediated down-regulation of miR-206, which further results in HDAC4 induction and consequent up-regulation of cyclin D1. The activation of AMPK by metformin inhibits TGF-β1-induced ASMCs proliferation, which is coupled to AMPK inhibition of miR-206/HDAC4/cyclin D1 pathway, particularly HDAC4 phosphorylation and expression. These findings provide important insights into TGF-β1 stimulation of ASMCs proliferation and highlight a novel mechanism whereby activation of AMPK may prevent/treat asthma by inhibiting airway remodeling.

TGF-β1 is a member of TGF-β superfamily and exerts its function by modulating a complicated intracellular network and autocrine signaling loops^24,25^. In the context of asthma, TGF-β1 is found to be elevated in airway, peripheral blood and bronchoalveolar lavage fluid (BALF) in asthmatic patients^4–6^ and contributes to airway remodeling by stimulating ASMCs proliferation^26^. With rapidly growing interest in the biological effect of miRNAs, we examined the cross-talk mechanisms between TGF-β1 and miRNA networks. MiR-206 is an important miRNA in regulation of myogenic differentiation^27^ and is also reported to play a great role in cancer cell migration and proliferation by targeting multiple genes^10,28^. The level of miR-206 is decreased in several types of cancer cell and highly proliferative cells^12,29,30^. Recently, miR-206 has been found to be down-regulated in the lung and blood of asthmatic patients and considered as a predictive biomarker for allergic and asthmatic status^13^. In addition, a study by Winbanks *et al*. has shown that miR-206 is decreased in primary muscle cells which display increased proliferation with treatment of TGF-β1^14^. Our study demonstrated that TGF-β1 inhibited miR-206 expression and then stimulated ASMCs proliferation by activation of Smad2/3. Overexpression of miR-206 suppressed TGF-β1-induced ASMCs proliferation, suggesting that reduction of miR-206 mediates TGF-β1 stimulation of ASMCs proliferation.

HDAC4 belongs to class II histone deacetylase family which regulates the balance of histone acetylation with histone acetyl-transferase (HAT)^31^. HDAC4 has been reported to play great roles in different biological processes involving cell differentiation, proliferation and migration^32^. Studies have demonstrated that up-expression of HDAC4 is closely associated with cell hyperplasia^33–36^. HDAC4 has been shown to promote cell proliferation by up-regulating cyclin D1 protein level^33,37–39^. Cyclin D1 is a member of cyclin protein family and is involved in regulating cell cycle progression. High expression of cyclin D1 drives the transition of cells from G1 to S phase, and promotes cells proliferation^40^. Accumulated studies have demonstrated that miR-206 suppresses HDAC4 expression by binding to HDAC4 3′-untranslated region^14,41,42^. Dai *et al*. have reported that inhibition of miR-206 increases HDAC4 protein level and promotes proliferation of satellite cells^43^. The present study indicated that TGF-β1 induced HDAC4 expression, which in turn increased cyclin D1 protein level and consequently stimulated ASMCs proliferation. Our results further exhibited that down-regulation of miR-206 mediated TGF-β1-induced elevation of HDAC4 in ASMCs.

Several studies have shown that activation of AMPK suppresses proliferation of a wide variety of cell types^44–46^. The present study indicated that miR-206/HDAC4/cyclin D1 signaling pathway contributed to TGF-β1-induced ASMCs proliferation, and activation of AMPK inhibited ASMCs proliferation by targeting on this pathway. Our results showed that activation of AMPK slightly increased miR-206 reduction but dramatically suppressed HDAC4 protein expression induced by TGF-β1. At the same time, we found that activation of AMPK also increased the phosphorylation of HDAC4. Previous studies have shown that activation of AMPK leads to HDAC4 inactivation by increasing HDAC4 phosphorylation in hepatocytes and myoblasts^21,47^. All these results suggest that activation of AMPK might largely target on HDAC4 by affecting its expression and phosphorylation to suppress TGF-β1-stimulated ASMCs proliferation. However, it has been shown that mTOR is involved in TGF-β1 induced proliferation in several types of cells, such as fibroblasts and cancer cells^48,49^, and activation of AMPK also inhibits these cells proliferation by suppressing mTOR activity^50,51^. Therefore, apart from miR-206/HDAC4/cyclin D1 signaling pathway, targeting on mTOR might also be involved in the inhibitory effect of AMPK on TGF-β1 induced ASMCs proliferation. Nevertheless, this speculation needs further verification.

Airway remodeling is closely associated with the morbidities of severe asthma^2^. The present study demonstrates that miR-206/HDAC4/cyclin D1 signaling pathway is involved in TGF-β1-induced ASMCs proliferation, suggesting that strategy targeting on this pathway might be a novel approach in the prevention and treatment of airway remodeling. Metformin, as one of representative member of biguanides and AMPK agonists, has been commonly used to treat type II diabetes with wide clinical experience and safety record^52^. Park *et al*. have found that activation of AMPK ameliorates airway remodeling in a murine model of chronic asthma^53^; together with our current findings, all these results suggest that activation of AMPK could be a novel strategy for the clinical treatment of airway remodeling. Yet, this remains to be verified by clinical trials.

## Materials and Methods

### Cell preparation and culture

Primary cultures of ASMCs from trachea and main bronchi of Sprague-Dawley rats (70–80 g) were isolated as previously described^54^. All animal procedures were performed in accordance with the Guide for the Care and Use of Laboratory Animals of Xi’an Jiaotong University Animal Experiment Center. All protocols used in this study were approved by the Laboratory Animal Care Committee of Xi’an Jiaotong University. Briefly, the trachea and main bronchi were rapidly removed from euthanized rats by CO_2_ overdose, washed in phosphate-buffered saline (PBS; 4 °C) and then dipped into Dulbecco’s Modified Eagle Medium (DMEM; Gibco) with 10% fetal bovine serum (FBS, Sijiqing, Hangzhou, China), 100 U/ml penicillin, and 100 μg/ml streptomycin. The epithelium and serosa were carefully stripped off using fine forceps and a surgical blade. Then the remaining tissue was cut into 0.5 mm pieces and placed into a culture flask and incubated at 37 °C in an atmosphere of 95% air and 5% CO_2_ till cells reaching 80% confluence. And cells were fed every 2–3 days and passaged by trypsinization using 0.25% trypsin (Invitrogen, Carlsbad, CA, USA). All experiments were performed using cells between passages 4 and 8. The purity and identity of ASMCs was determined by immunostaining with α-smooth muscle actin (α-SMA; Boster, Wuhan, China). Fluorescence microscope images indicated that cells contained more than 90% of ASMCs (data not shown here). Before each experiment, cells were incubated in 1% FBS-DMEM overnight to minimize serum-induced effects on cells. TGF-β1 (PeproTech, Rocky Hill, NJ, USA, distillated water as vehicle) was used to stimulate cell proliferation. SB431542 (Cell Signaling Technology, Beverly, MA, USA, DMSO as vehicle), a specific inhibitor of the TGF-β type 1 receptors ALK5, was used to block Smad2/3. Metformin was purchase from Bristol-Myers Squibb biopharmaceutical company (DMEM as vehicle).

### Cell proliferation assay

To examine ASMCs proliferation, the rate of BrdU incorporation was determined using BrdU ELISA Kit (Maibio, Shanghai, China) according to the manufacturer’s instructions. Briefly, cells were seeded into 96-well plate at 5 × 10^3^ cells per well, allowing to adhere for at least 24 h, and serum starved overnight (1% FBS in DMEM) before the start of experiments. After different treatments, BrdU labeling reagent was added to the wells and incubated for 2 h at 37 °C. Then, cells were denatured with FixDenat solution for 30 min at room temperature, and followed by incubating with anti-BrdU mAbs conjugated to peroxidase for 90 min at room temperature. After removing antibody conjugate, substrate solution was added for reaction of 10 min. The absorbance at 370 nm was determined with a microplate reader (Bio-Rad, Richmond, CA, USA). The blank corresponded to 100 μl of culture medium with or without BrdU.

### Oligonucleotide synthesis and transfection

The miRNA-206 mimic, miRNA-206 inhibitor, siRNA-HDAC4 (siHDAC4), siRNA-AMPK α2 (siAMPK α2) and negative control oligonucleotides were synthesized by GenePharma (Shanghai, China). ASMCs were seeded in 6-well plates at a density of 2 × 10^5^ cells/well and cultured till reaching 30–50% confluence. Then cells were transfected with siRNA or miRNA (100 nM) using Lipofectamine^TM^ 2000 (Invitrogen) according to the manufacturer’s instructions. After transfection, cells were maintained for 24 h in DMEM containing 10% FBS, 100 IU/ml penicillin, and 100 μg/ml streptomycin. Effects of siRNA and miRNA transfection were determined using western blotting and qRT-PCR respectively.

### Quantitative real-time polymerase chain reaction (qRT-PCR)

Total RNA including miRNA fraction was collected from ASMCs using a TRIzol-based extraction protocol (Invitrogen). Isolated RNAs were polyadenylated using the Thermo Scientific RevertAid First Strand cDNA Synthesis Kit (Logan, UT, United States). cDNA synthesized was used to perform quantitative PCR on an IQ™5 Multicolor Real-Time PCR Detection System (Bio-Rad) using the SYBR Green Real-time PCR Master (TaKaRa, Tokyo, Japan). Primers specific for miR-206 and U6 small nuclear RNA were purchased from Sangon Biotech (Shanghai, China), the following primer sets were used: rat miR-206, RT primer, 5′-GTCGTATCCAGTGCAGGGTCCGAGGTATTCGCACTGGATACGACCCACAC-3′, Forward, 5′-GCGGCGGTTCACAGTGGCTAAG-3′, Reverse, 5′-ATCCAGTGCAGGGTCCGAGG-3′; U6, RT primer, 5′-GTCGTATCCAGTGCAGGGTCCGAGGTATTCGCACTGGATACGACAAAATA-3′, Forward, 5′-AGAGAAGATTAGCATGGCCCCTG -3′, Reverse, 5′-ATCCAGTGCAGGGTCCGAGG-3′. The fold increase relative to control samples was determined by 2^−ΔΔCt^ method. The miRNA expression was normalized to U6 small nuclear RNA. Amplification was performed at 95 °C for 30 s, followed by 40 cycles of 95 °C for 5 s and 58 °C for 30 s and 72 °C for 30 s.

### Assessment of AMPK activity

AMPK activity was detected with an AMPK ELISA kit (MLBIO Biotechnology Co. Ltd, Shanghai, China). Briefly, ASMCs were seeded in 6-well plates at a density of 2 × 10^5^ cells/well and received different treatments. After treatment, cells were lysed and centrifuged. Then, the supernatant was collected and AMPK activity assay was analyzed according to manufacturer’s instructions. A standard curve was generated using the standards and their respective absorbance values at 450 nm. The AMPK activity of samples was calculated based on the absorbance values at 450 nm and the standard curve.

### Immunoblotting

Cells were lysed in RIPA Lysis Buffer (50 mM Tris PH 7.4, 150 mM NaCl, 1% NP40, 0.5% Sodium-deoxycholate, 0.1% SDS, 1 mM EDTA, 1 mM phenylmethylsulfonyl fluoride, 1 mM Na_3_VO_4_, 1 mM NaF and proteinase inhibitors). Lysates were centrifuged at 13000 rpm for 10 min at 4 °C, and supernatant was collected as sample protein and protein concentration was determined with the BCA protein assay kit (Pierce, Rockford, IL, USA). Protein was separated on a SDS-PAGE gel and transferred to a Trans-Blot Nitrocellulose membrane (Bio-Rad). Polyclonal antibody against total-AMPK α (Cell Signaling Technology, Beverly, MA, USA, 1:1000 dilution) and AMPK α2 (Proteintech Group, Chicago, IL, USA, 1:1000 dilution) and monoclonal antibodies against total-Smad2/3 (Abcam, Cambridge, MA, USA, 1:1000 dilution), β-actin (Santa Cruz Biotechnology, USA, 1:500 dilution), phospho-Smad2/3, HDAC4, cyclin D1, phospho-AMPK α and phospho-HDAC4 (Cell Signaling Technology, 1:1000 dilution) were used following manufacturer’s protocols. Horseradish peroxidase-conjugated goat anti-rabbit or anti-mouse IgG was determined as the secondary antibody (Santa, 1:2000 dilution). Membranes were visualized on a ChemiDoc XRS system and analyzed using Quantity One software (Bio-Rad).

### Statistical analysis

Data are presented as mean ± SD. Differences among groups were analyzed using one-way analysis of variance followed by Tukey post hoc test. P < 0.05 was considered statistically significant.

## Electronic supplementary material

Supplementary Material
