# Supplementary material for: Activation of AMPK inhibits TGF-β1-induced airway smooth muscle cells proliferation and its potential mechanisms
Source: Sci Rep. 2018 Feb 26;8:3624. doi: 10.1038/s41598-018-21812-0 (PMC5827654; doi:10.1038/s41598-018-21812-0)
Supplement: Supplementary file 1 — Supplementary Material [file 41598_2018_21812_MOESM1_ESM.pdf]

Activation of AMPK inhibits TGF-β1-induced airway smooth muscle cells proliferation and its potential mechanisms

Yilin Pan[1], Lu Liu[1], Shaojun Li[1], Ke Wang[2], Rui Ke[1], Wenhua Shi[1], Jian Wang[1], Xin Yan[1], Qianqian Zhang[1], Qingting Wang[1], Limin Chai[1], Xinming Xie[1] and Manxiang Li[1,*]

1 Department of Respiratory and Critical Care Medicine, the First Affiliated Hospital of Xi'an Jiaotong University, Xi'an710061, P.R. China

2 School of Pharmacy, Xi'an Jiaotong University, Xi'an710061, P.R. China

Corresponding Author's information:

Manxiang Li

Department of Respiratory and Critical Care Medicine, the First Affiliated Hospital of Xi'an Jiaotong University

No.277, West Yanta Road,

Xi'an, Shaanxi, P.R. China 710061

Telephone: +86-029-85324053

E-mail address: manxiangli@hotmail.com

Supplementary Figure S1

Figure 1c

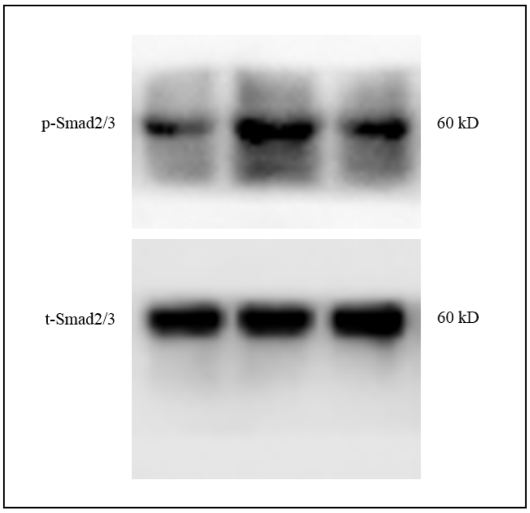

Figure 2c

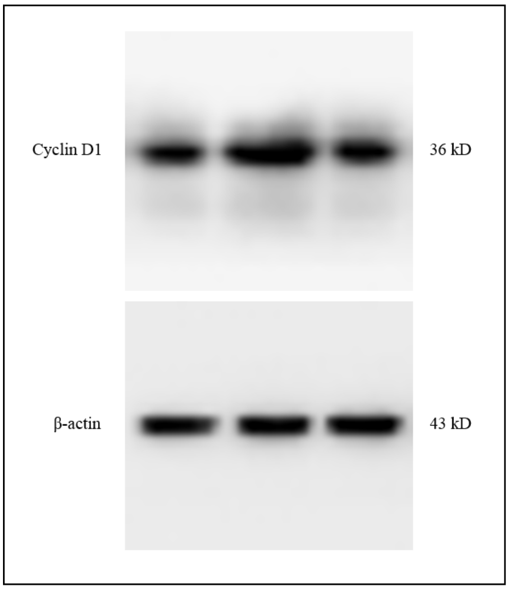

Figure 2b

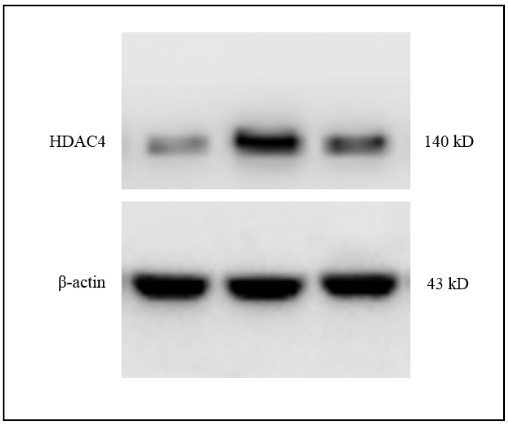

Fig. S1. Full-width of membrane with a protein marker besides the each box of the original blots used in Figures 1&2. The molecular size is shown as indicated. Panels shown in this supplemental figure correspond to those in the main article.

Supplementary Figure S2

Figure 3c

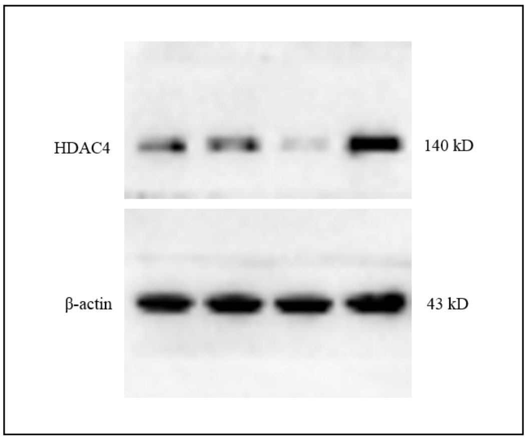

Figure 3d

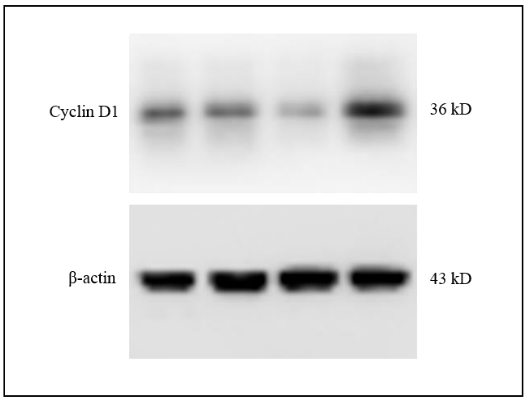

Figure 3e

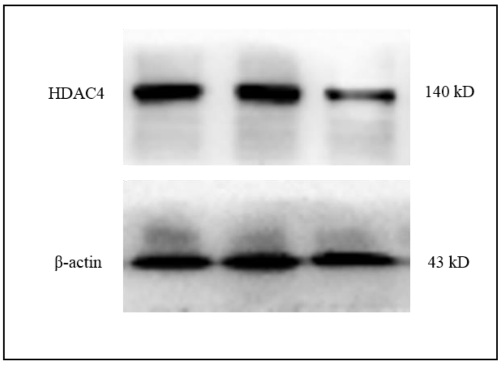

Figure 3f

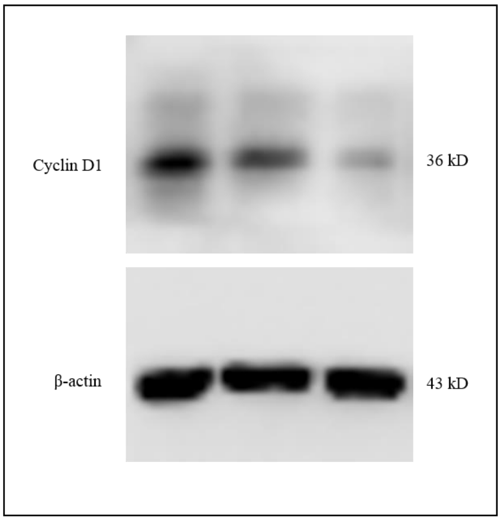

Fig. S2. Full-width of membrane with a protein marker besides the each box of the original blots used in Figures 3. The molecular size is shown as indicated. Panels shown in this supplemental figure correspond to those in the main article.

Supplementary Figure S3

Figure 4a

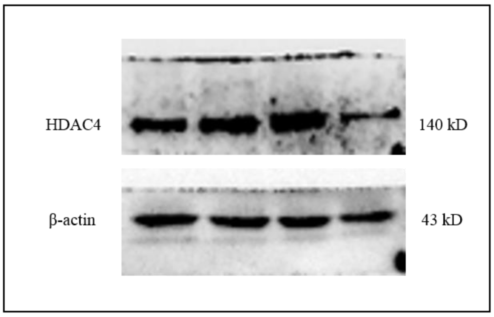

Figure 4b

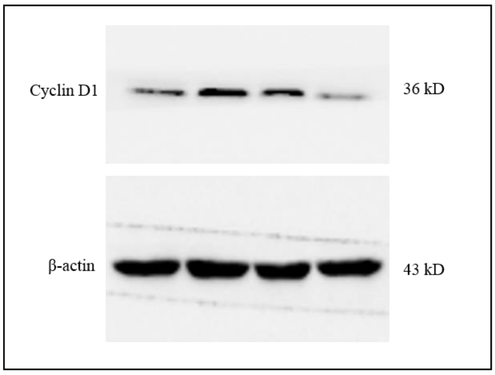

Figure 4d

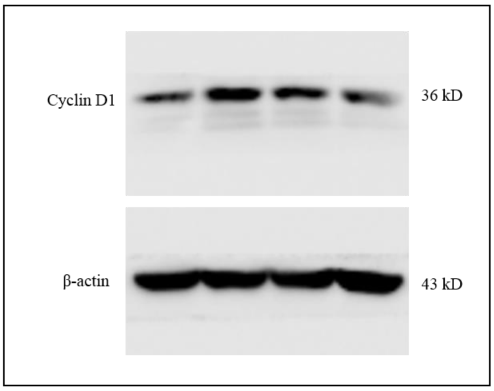

Fig. S3. Full-width of membrane with a protein marker besides the each box of the original blots used in Figures 4. The molecular size is shown as indicated. Panels shown in this supplemental figure correspond to those in the main article.

Supplementary Figure S4

Figure 5a

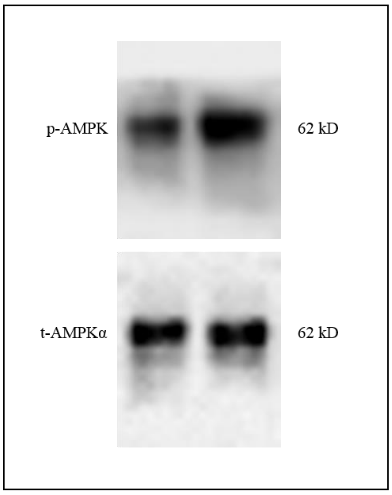

Figure 5c

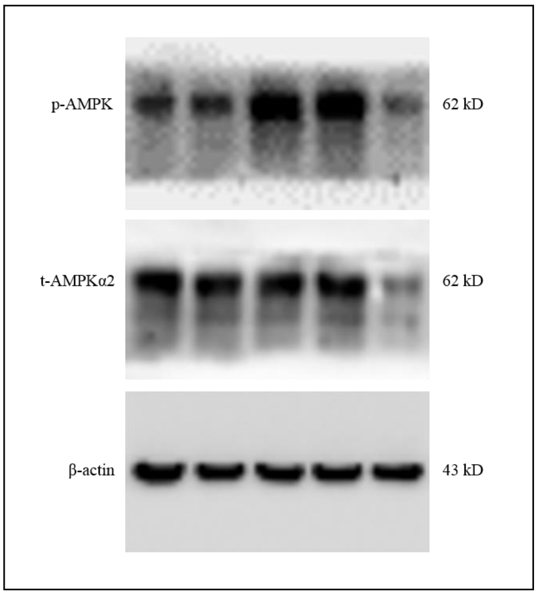

Figure 6a

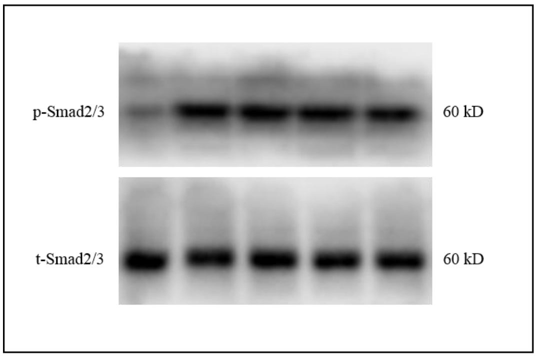

Figure 6c

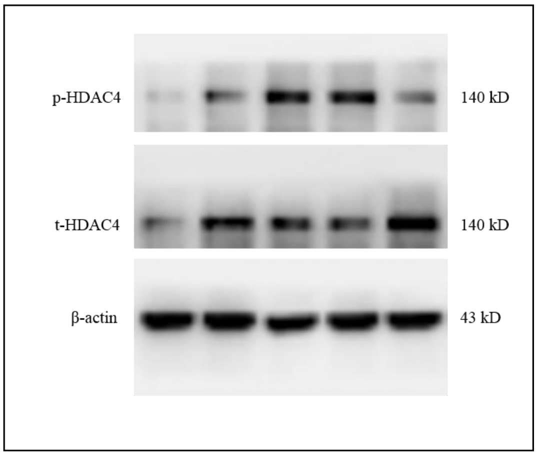

Figure 6d

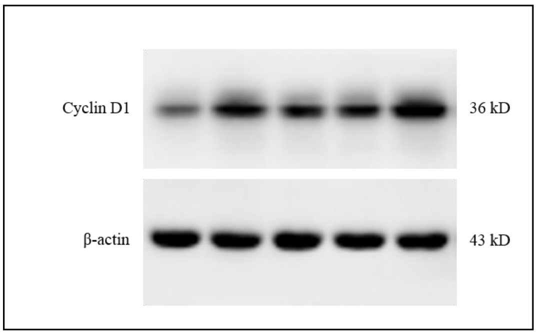

Fig. S4. Full-width of membrane with a protein marker besides the each box of the original blots used in Figures 5&6. The molecular size is shown as indicated. Panels shown in this supplemental figure correspond to those in the main article.
